# Optimization of Conditions for Extraction of Polyphenols and the Determination of the Impact of Cooking on Total Polyphenolic, Antioxidant, and Anticholinesterase Activities of Potato

**DOI:** 10.3390/foods7030036

**Published:** 2018-03-09

**Authors:** Imen Laib, Malika Barkat

**Affiliations:** Laboratoire BIOQUAL, Institut de la Nutrition, de l’Alimentation et des Technologies Agro-Alimentaires (I.N.A.T.A.A.), Université Frères Mentouri-Constantine 1, Route de Ain El-Bey, 25000 Constantine, Algeria; barkat.inataa@yahoo.fr

**Keywords:** potato, polyphenols, experimental design, LC-MS/MS, antioxidant activity, anticholinesterase activity

## Abstract

In this work we optimized the cooking and extraction conditions for obtaining high yields of total polyphenols from potato and studied the effect of three domestic methods of cooking on total phenols, antioxidant activity, and anticholinesterase activities. The optimization of the experiment was carried out by the experimental designs. The extraction of the polyphenols was carried out by maceration and ultrasonication. Determination of the polyphenols was performed by using the Folin-Ciocalteau reagent method. The antioxidant activity was evaluated by three methods: 1,1-diphenyl-2-picryl-hydrazyl (DPPH), 2,2′-azino-bis(3-ethylbenzothiazoline-6-sulphonic acid) (ABTS), and CUPRAC(Cupric reducing antioxidant capacity), the anticholinesterase activity was evaluated by the method of Elmann. The optimum of total phenolic obtained was: 4.668 × 10^4^, 1.406 × 10^4^, 3357.009, 16,208.99 µg Gallic Acid Equivalent (GAE)/g of dry extract for crude potato, steamed potatoes, in boiling water, and by microwave, respectively. The three modes of cooking cause a decrease in the total polyphenol contents, antioxidant and anticholinesterase activities.

## 1. Introduction

The appearance and progression of some diseases are clearly due to many lifestyle factors. Nutrition is one of the most important determinants of health, and increasing evidence suggests that diets rich in vegetables and fruits can prevent many diseases [1]. Although the mechanisms of these effects have not been fully elucidated, the presence of secondary metabolites, commonly referred to as phytochemicals, in fruits and vegetables may play a major role [2,3].

Many studies show that the majority of phytochemicals have antioxidant [4,5] and anti-inflammatory [6,7] properties. These activities give them pharmacological properties that may prevent, and possibly even treat, different diseases [8]. Among these abundant compounds in vegetables, phenolic compounds are the most important group of natural antioxidants [9].

Vegetable phenolic compounds have been of great interest because of growing evidence of their beneficial effect on human health. These compounds may have complementary mechanisms of action, including stimulation of the immune system, reduction of platelet aggregation, modulation of lipid and hormone metabolism, and antioxidant, antibacterial, antimutagenic, and antiangiogenic effects, reduction of the initiation of tumors, and the induction of apoptosis [10].

Recent studies have shown the effect of polyphenols against Alzheimer’s disease [11]. These compounds are classified as cholinesterase inhibitors, which are valuable approaches for the treatment of neurodegenerative diseases, because of their role in the maintenance of the neurotransmitter acetylcholine [12]. Neurodegenerative disorders are characterized by reduced cholinergic activity in the brain [13]. The acetylcholinesterase and butyrylcholinesterase enzymes cause the hydrolysis of acetylcholine; therefore, inhibition of these enzymes by polyphenols plays a key role in improving cholinergic activity and preventing Alzheimer’s disease [14]. The potato (*Solanum tuberosum* L.) is a tuber that contains different bioactive compounds, such as starch, dietary fiber, amino acids, minerals, vitamins, and phenolic compounds. Phenolic compounds are synthetized by the potato plant as a protection response from bacteria, fungi, viruses, and insects. Potato is considered as a food rich in polyphenols, and these antioxidant compounds protect the body’s cells from damage caused by free radicals. The phenolic content of the potato differs considerably depending on the variety, the growing and climatic conditions, and the methods of analysis used [15]. However, potato must be cooked for consumption. According to the recipes and culinary traditions of the various countries, household cooking encompasses a variety of processes, such as boiling, frying, steaming, roasting, and microwaving [16]. Although the cooking of potato has beneficial effects, including the destruction of microorganisms, the inactivation of anti-nutritional factors, the improvement of the digestibility of food, the bioavailability of nutrients, and the formation of desired compounds such as flavoring compounds and antioxidants, it also has negative effects, such as changing the structure of vegetables, leading to nutritional effects. On the other hand, the treatment can damage the quality of food, resulting in undesirable consequences, such as the loss of certain nutrients due to chemical reactions and the formation of undesirable compounds (for example, acrylamide) [10].

Polyphenols of potato include many compounds that behave differently during heat treatment. In recent years, many studies have focused on the various cooking effects on the total polyphenol content of vegetables and their biological activities. Some authors confirm the hypothesis of the reduction of polyphenol content after cooking, while others have shown a positive effect on these compounds. Changes in the content of phenolic compounds by heat treatment of potatoes have been the subject of numerous studies [17,18,19,20,21,22], but optimization of cooking and extraction conditions from potato have not yet been studied.

Optimization of conditions of the cooking process can help the consumer and agri-food industry to improve the nutritional quality of cooked vegetables and avoid or minimize losses of polyphenols. 

There is no single, standardized method for extracting polyphenols, particularly because of their diversity. According to several studies the extraction efficiency is, therefore, dependent on numerous factors, like the process conditions, the solvent concentration, the particle size, the nature of the solvent, and the extraction time [23]. Then to extract all the polyphenols from the plant matrix, it is necessary to optimize the extraction conditions.

The objective of this work is to select the factors that have a proven influence on the polyphenol content of raw and cooked potato, using screening experimental design which quantify and classify the effects of each of the factors, After looking for the effect of each parameter, we want to optimize the extraction conditions of the polyphenols of the extraction conditions by the central composite design and determine the impact of cooking on total phenolic, antioxidant, and anticholinesterase activities of potato.

## 2. Materials and Methods

### 2.1. Plant Material

White potato, Spunta variety (1 kg), was harvested from Benibechir, Skikda, Algeria.

### 2.2. Cooking Vegetables

Potatoes are washed and all non-edible parts removed, peeled, and then they are cut into small pieces of homogeneous shapes and cooked by: boiling, steaming, and microwaving. The cooking waters were recovered. 

### 2.3. Extraction

The polyphenols of raw and cooked potatoes are extracted by maceration and ultrasonication. The raw and cooked raw potatoes were mashed and then extracted with methanol in a beaker and then placed in an ultrasonicator. Then, the extracts were filtered, the mashed potato were re-extracted three times, and the combined extracts were used for the experiments. The solvent was evaporated by a rotary evaporator. The cooking water is analyzed directly, and it requires no treatment other than filtration and evaporation under vacuum.

### 2.4. Optimization of Cooking and Extraction Conditions

In this work, we attempt to optimize the conditions of cooking and extraction of phenolic compounds to obtain a maximum of polyphenols in the extracts of raw and cooked potatoes. For this, an experimental design has been developed. The methodology used is divided into two stages: a factor screening by the Plackett–Burman design and an optimization by exploiting surfaces plots.

#### 2.4.1. Screening Factors

Plackett–Burman experimental design was chosen to perform the screening of factors (Table 1). Plackett–Burman’s experimental design is used to select the most influential factors on the Y response (total phenolic) where interactions are negligible or thought to be negligible [24].

Factors of cooking (volume of water, time, and cooking temperature (in the case of boiling and steam cooking); volume of water used, time, and power (in the case of microwave cooking)), factors of extraction (the concentration of the extraction solvent, the maceration time, the ultrasonication time), and their levels (minimum (−1) and maximum (+1)) were chosen by reference to preliminary analyses and bibliography. Grinding is also a factor that has a significant effect on the polyphenol content of raw and cooked vegetables according to several studies [25].

The mathematical model is a model without interaction:

*Y*= b0 + b1*X*1 + b2*X*2 + b3*X*3+ b4*X*4 + b5*X*5 + b6*X*6 + *ɛ* (1)

where *Y* is the yield of total phenolics; b0 is the theoretical mean value of the response; b1, b2, b3, b4, b5, b6 are the main effects of the factors *X*1, *X*2, *X*3, *X*4, *X*5, *X*6, respectively; and *ɛ* is the error term [26].

The model is statistically significant when the coefficient of determination is close to 100% and its probability of nullity α is less than 0.05%.

A factor has a significant effect on total phenolic when its probability of nullity α is less than 0.05%.

#### 2.4.2. Optimization of Responses

A central composite experiment design was used to optimize the effects of factors selected by the screening plan on the response of the noted polyphenol content. This experiment design is based on the surface plot method. It consists in modeling the results in the form of polynomial functions of the second degree, which is a quadratic model [27]. We have assigned for each coded variable (*X*i) three levels: −1, 0 and +1. ±α represents the extreme values (max, min). For the design to be orthogonal and rotatable, the value of α was fixed by the software STATISTICA 7.0 (StatSoft, Inc., Tulsa, OK, USA) at 1.414.

The mathematical model of the second degree of composite centered design is:

*Y* = a0 + Σai*X*i + Σaii*X*i2 + Σaij*X*i*X*j (2)

ai: regression coefficients of linear effects; aii: regression coefficients of quadratic effects; *X*i and *X*j: coded experimental variables [27].

### 2.5. Determination of Total Polyphenols (TP)

The polyphenols are determined using the Folin–Ciocalteu reagent method [28].

### 2.6. LC-MS/MS Analysis

Analysis of the LC-MS/MS phenolic compounds was performed using a Shimadzu Nexera model CLUHP coupled to a tandem MS instrument (Shimadzu, Kyoto, Japan). The liquid chromatography is equipped with LC-30AD binary pumps, a DGU-20A3R degasser, a UDC CTO-10A column, and a SIL-30AC sampler. The chromatographic automatic separation was performed on an ODS-4 reverse phase C18 analytical column (150 mm × 4, 6 mm, 3 μm). The temperature of the column was set at 40 °C. The elution gradient was composed of amobile phase A (water, 5 mM ammonium formate, and 0.1% formic acid) and aphase mobile B (methanol, 5 mM ammonium formate, and 0.1% formic acid). The gradient program with the following proportions of solvent B was applied (*t* (min), %B) (0.40) (20.90), (23.99, 90), (24.40). The solvent flow rate was maintained at 0.5 mL/min and the injection volume was adjusted to 4 μL. The MS detection was performed using a Shimadzu LCMS 8040 quadrupole model LCMS mass spectrometer equipped with an ESI source operating in both positive and negative ionization modes. LC-MS/MS data was collected and processed by Lab Solutions software (Shimadzu, Kyoto, Japan). Multiple reactions (MRM) mode was used to quantify the analytes: the compounds tested were assayed in two or three transitions for each component, the first for quantitative purposes and the second and/or third for the confirmation.

In this study, twenty-four phenolic compounds (flavonoids, flavonoid glycosides, phenolic acids, phenol aldehyde, coumarin) and three non-phenolic organic acids that are widely distributed in food materials were qualified and quantified in potato

### 2.7. Antioxidant Activity

Evaluation of the antioxidant activity is carried 1,1-diphenyl-2-picryl-hydrazyl (DPPH), 2,2′-azino-bis(3-ethylbenzothiazoline-6-sulphonic acid) (ABTS), and cupric reducing antioxidant capacity (CUPRAC) methods.

#### 2.7.1. DPPH Method

The evaluation of the antioxidant activity was carried out by the method of Blois [29].

#### 2.7.2. ABTS Method

The evaluation of the antioxidant activity was carried out by the ABTS free radical scavenging method described by Re et al. [30].

#### 2.7.3. CUPRAC Method

The evaluation of the antioxidant activity was carried out by the method of Özyürek et al. [31].

### 2.8. Anticholinesterase Activity

Anticholinesterase activity is carried out according to the method of Ellman [32].

### 2.9. Statistical Analysis

The means plus or minus the standard deviation of the three replicates of determination of total polyphenol content, evaluation of antioxidant activity, and anticholinesterase activity, as well as graphic representations, were performed with Excel 2013 (Microsoft Excel Version 3. 2013, Microsoft Corp., Redmond, WA, USA). Means were compared by single factor analysis of variance (ANOVA) analysis followed by a post-hoc Tukey test using STATISTICA 7.0 software (StatSoft, Inc., Tulsa, OK, USA). The superscript letters a, b, c, d, and e indicate a significant difference at the 0.05 significance level. The generation of the test matrices, the analysis of the results of the two experimental designs (Plackett–Burman and the central composite design) are generated with Minitab 17 software (Minitab Inc., State College, PA ,USA). The generation of surface plots and the optimization of the factors were made by STATISTICA 07.0 software (StatSoft, Inc., Tulsa, OK, USA).

## 3. Results and Discussion

### 3.1. Experiment Design

#### 3.1.1. Screening Design

**Figure 1:**
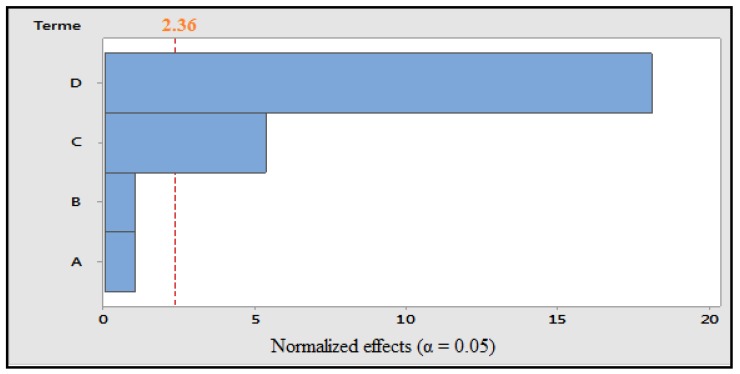
Pareto diagram of normalized effects (α = 0.05). A: concentration of solvent; B: grinding; C: maceration time; and D: ultrasonication time.

Raw potato: Design analysis shows that the coefficient of determination values and the adjusted coefficient of determination are close to 100%. The probability is less than 0.05, which indicates that the mathematical model of this plan is significant. The *p* values obtained for each factor showed that the maceration time and the ultasonication time had significant effects on the response total phenolic (Figure 1). The polynomial equation for the response is:

TP = 14685 + 242A + 242B − 1259C + 4257D(3)

**Figure 2:**
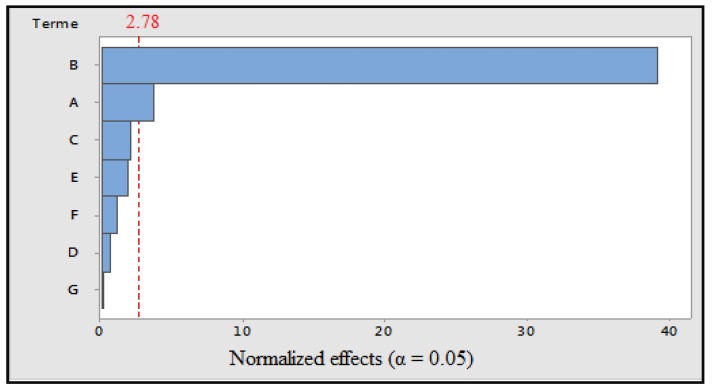
Pareto diagram of normalized effects (α = 0.05). A: cooking time; B: cooking temperature; C: volume of cooking water; D: concentration of solvent; E: maceration time; F: ultrasonic time; and G: grinding.

Steaming: Analysis of the *p* values shows that cooking time and the cooking temperature had a significant effect on the response (Figure 2). The coefficient of determination indicates that 99.3% of the response variability could be explained by the model. The polynomial model equation is:

TP = 6049 + 531A − 5530B + 291C − 92D − 274E + 169F − 27G(4)

**Figure 3:**
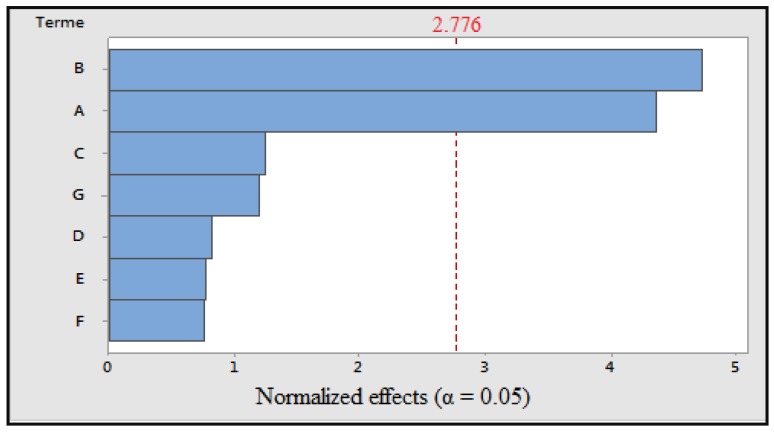
Pareto diagram of normalized effects (α = 0.05); A: cooking time; B: cooking temperature; C: volume of cooking water; D: concentration of solvent; E: maceration time; F: ultrasonic time; and G: grinding.

Boiling: The coefficient of determination is close to 100% and *p* is less than 0.05, indicating that the model is significant. Among the variables tested, the cooking time and the cooking temperature had a significant effect on the response (Figure 3). The polynomial equation for the response is:

TP = 1408 − 567A + 615B + 163C + 107D + 101E − 99F + 156G(5)

**Figure 4:**
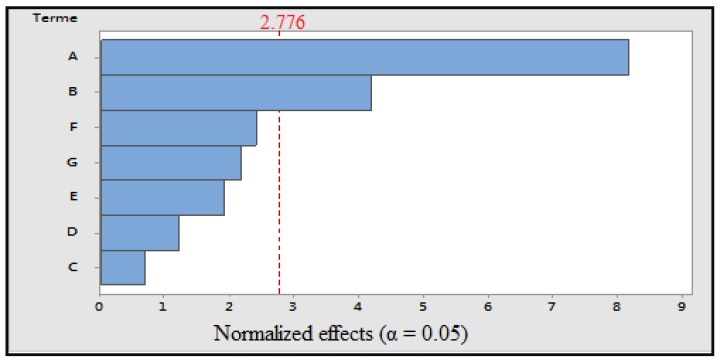
Pareto diagram of normalized effects (α = 0.05); A: cooking time; B: power; C: volume of cooking water; D: concentration of solvent; E: maceration time; F: ultrasonic time; and G: grinding.

Microwaving: The coefficient of determination values and the adjusted coefficient of determination are close to 100% and the value of *p* obtained for this model is less than 0.05, which indicates that the model is significant. The variables having a significant effect on the response are cooking time and power (Figure 4). The polynomial equation for the response is:

TP = 10224 + 5058A + 2593B − 425C + 749D + 1181E − 1497F − 1348G(6)

#### 3.1.2. Central Composite Design

##### Raw Potato

After looking for the effect of each parameter, we want to optimize the extraction conditions of the polyphenols. The level of polyphenols depends on two factors of *m* = 2 factors (maceration time and ultrasonication time).

Both the coefficient of determination and the adjusted coefficient are close to 100% and the *p* value for the model was 0.000, indicating that the model was extremely significant. The quadratic polynomial equation is:

TP = 14626 + 36.85A − 745B + 0.00591AA + 22.03BB − 1.331AB(7)

The linear terms (maceration time (A), ultrasonication (B)), the quadratic terms (square of maceration time (AA) and ultrasonication time (BB) and the interaction between the two-factor (AB) were extremely significant.

**Figure 5:**
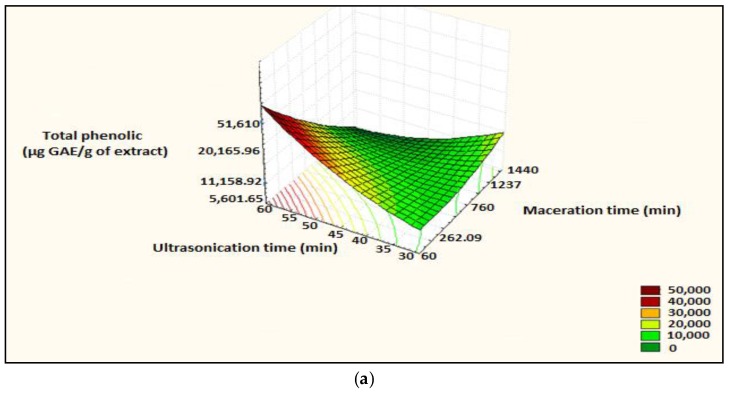

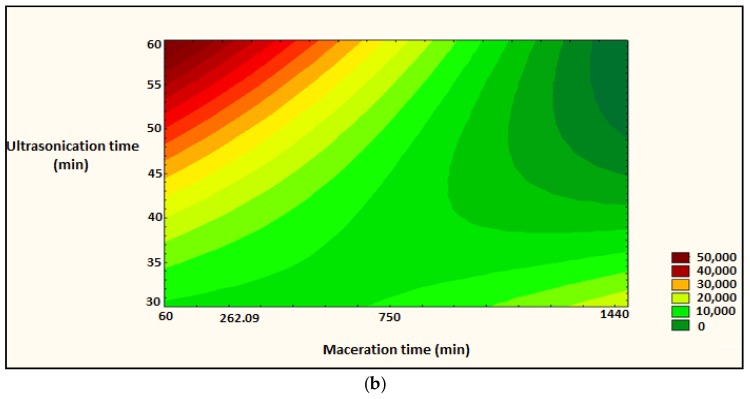
Surface plot and iso-response curve of polyphenols as a function of the two factors maceration time and ultrasonication time, (**a**): surface plot; (**b**): iso-response curve.

Surface plot illustrating the evolution of the response according to the levels of maceration time and ultrasonication was plotted (Figure 5).

**Figure 6:**
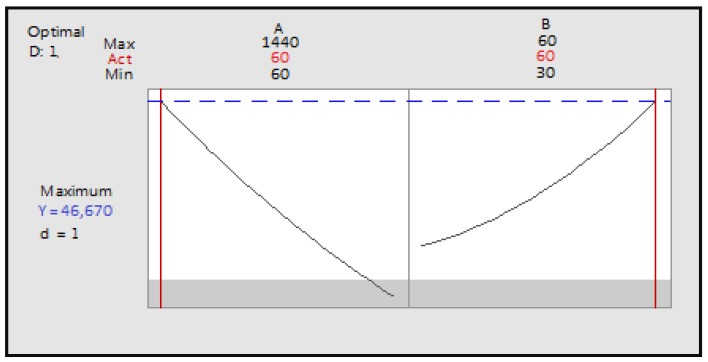
Optimization of the two factors maceration time and ultrasonication time as a function of desirability; A: maceration time; B: ultrasonication time.

The maximum accuracy is obtained for a desirability close to 1. Prediction of optimal conditions was: maceration time: 60 min, and ultrasonication time, 60 min, and predicted polyphenol content is 4.667 × 10^4^ µg Gallic Acid Equivalent (GAE)/g of extract (Figure 6).^.^

The results of the optimization are confirmed by the determination of the total polyphenols with the factors selected at the optimum point (Table 2). There is not a significant difference between measured and predicted values which confirms the validation of the model.

##### Steaming

Factors selected by the screening design (cooking time and cooking temperature) are optimized. The *p*-value for the model was 0.0007, indicating that the model was highly significant. Linear terms (cooking time (A), and cooking temperature (B) and the quadratic terms (square of cooking time (AA) and square of cooking temperature (BB) were extremely significant. Interaction between the two factors (AB) is not significant. The coefficient of determination value is 94.79%.

The polynomial equation is:

TP = 524 + 1961A + 2162B + 5527AA + 3629BB − 391AB(8)

**Figure 7:**
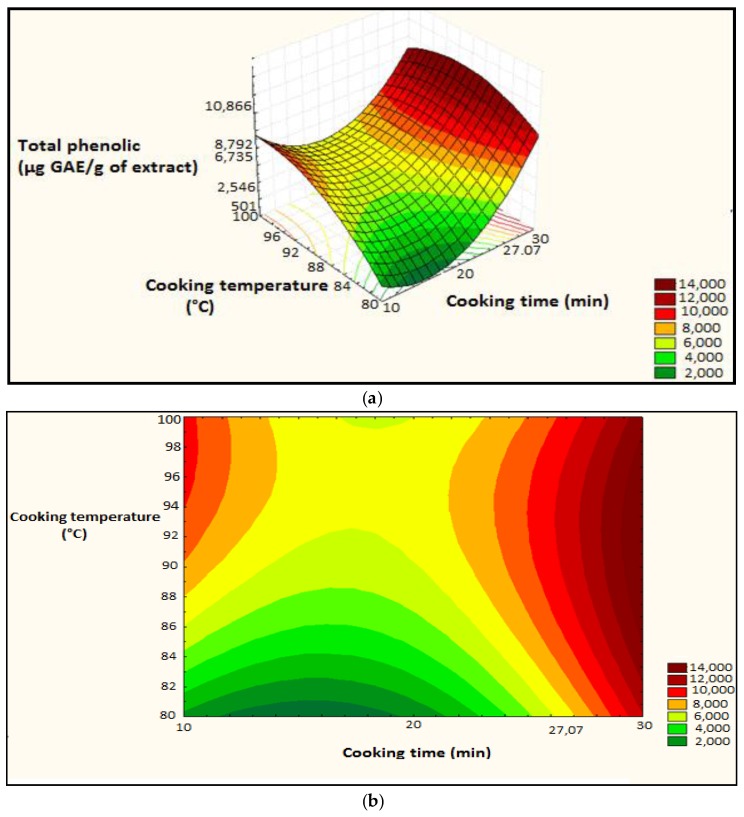
Surface plot and iso-response curve of polyphenols as a function of the two factors cooking time and temperature, (**a**): surface plot; (**b**): iso-response curve.

The surface plot and isoresponse curve were plotted according to the two factors (Figure 7).

**Figure 8:**
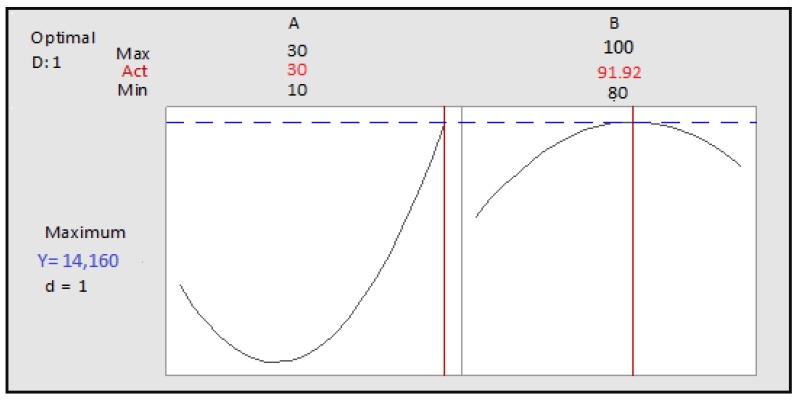
Optimization of the two cooking time and temperature factors as a function of desirability; A: Cooking time; B: Cooking temperature.

The optimum conditions were set as follows: cooking temperature: 91.9192 °C and the cooking time: 30 min. The predicted polyphenol content is 1.416 × 10^4^ µg GAE/g of extract (Figure 8).

The results of the optimization are confirmed by the determination of the total polyphenols with the factors selected at the optimum point (Table 3). There is not a significant difference between measured and predicted values, which confirms the validation of the model.

##### Cooking in Water

The two factors of cooking time and cooking temperature are optimized. The coefficients of determination obtained are high, indicating that the model is significant. Therefore, time (A) and temperature (B) significantly affected the total polyphenol content. The second-order effect of cooking time (AA) and the second-order effect of cooking temperature (BB) significantly affected the responses. Analysis of the interactions between the two factors showed that AB had a significant effect on the response.The quadratic polynomial equation is:

TP = 917.4 − 483.9A + 153.9B + 275.6AA + 472.0BB + 546.4AB(9)

**Figure 9:**
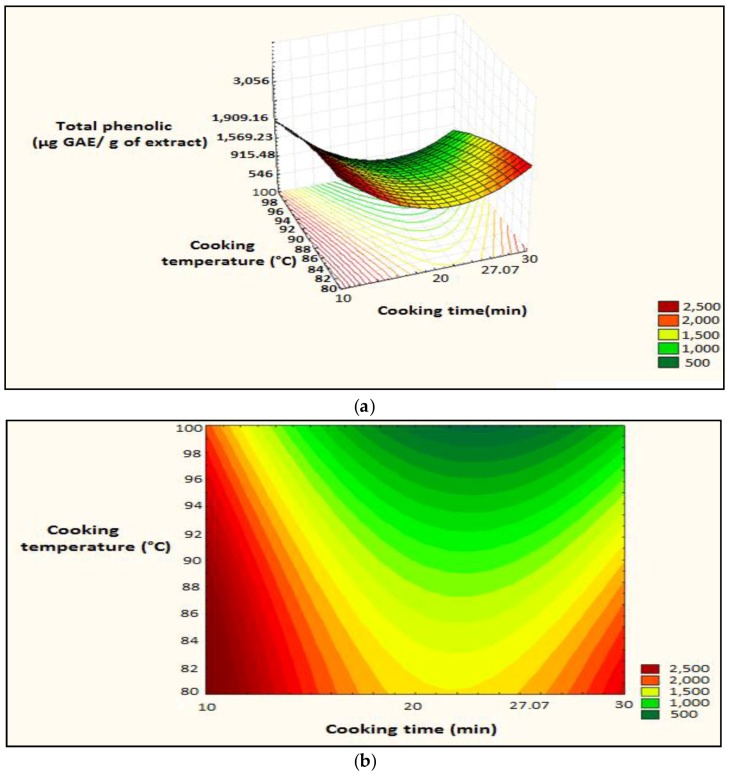
Surface plot and iso-response curve of polyphenols as a function of both time and temperature cooking factors, (**a**): surface plot; (**b**): iso-response curve.

The surfaceplot illustrating the evolution of the response as a function of the levels of the two selected factors was plotted (Figure 9).

**Figure 10:**
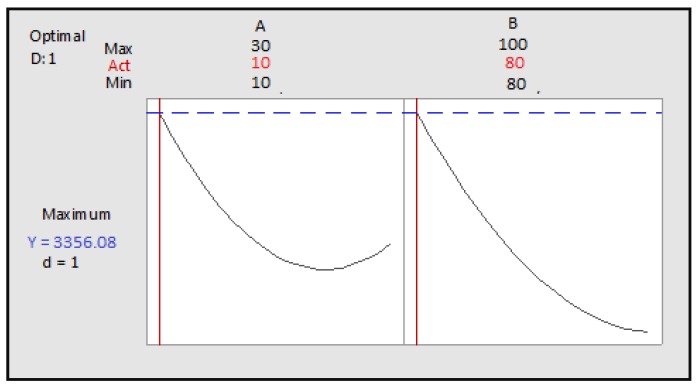
Optimization of two cooking time and temperature factors according to desirability; A: cooking time; B: cooking temperature.

The optimum conditions for achieving the best polyphenol content are: time: 10 min and temperature: 80 °C, and predicted polyphenol content is 3356.08 GAE/g of extract (Figure 10).

The optimum conditions obtained were applied to the laboratory to confirm the validity of the model (Table 4). There is no significant difference between the measured and predicted values, which confirms the validation of the model.

##### Microwave Cooking

The two factors cooking time and power factors were used for optimizing the total polyphenol response. The probability obtained for this model is <0.0001 which indicates that the mathematical model corresponds to the response. The coefficients of determination obtained are high, indicating that the model is significant. Therefore, time (A) and power (B) significantly affected the total polyphenol content. The second-order effect of cooking time (AA) significantly affected the response, while the second-order effect of power (BB) did not show any significant difference. Analysis of the interactions between the two factors showed that AB had a significant. The polynomial equation obtained for the response is:

TP = 14570 + 4418A + 1803B − 3050AA − 373BB − 2344AB(10)

**Figure 11:**
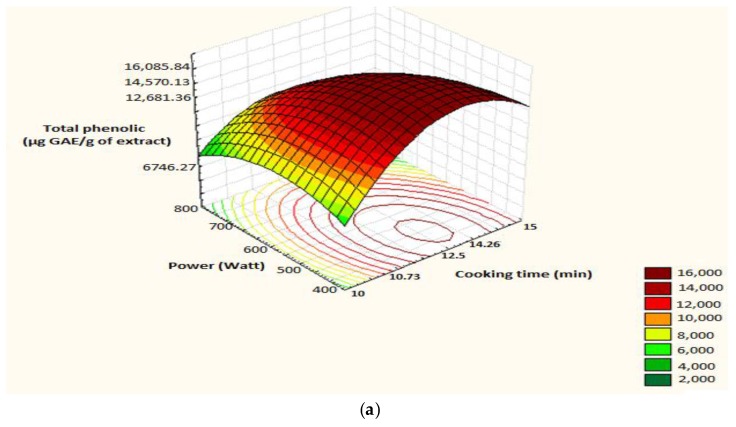

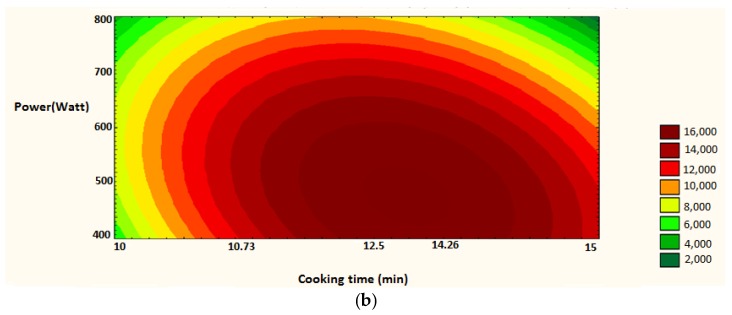
Surface plot and iso-response curve of polyphenols as a function of both factors time of cooking and microwave power. (**a**): surface plot; (**b**): iso-response curve.

The surface plot and iso-response curve are shown in Figure 11.

**Figure 12:**
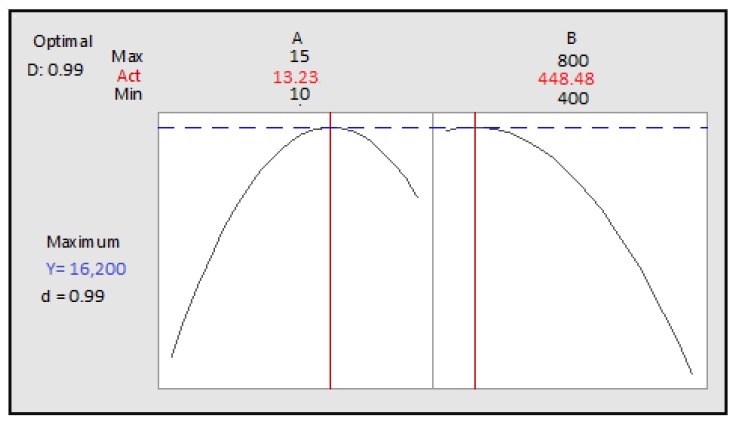
Optimization of two cooking time and power according to desirability; A: cooking time; B: power.

The optimum conditions obtained are as follows: cooking time: 13.23 min, power: 448.48 Watts (Figure 12), and the predicted polyphenol content is 16,210 µg GAE.

The optimum values obtained were verified experimentally (Table 5). There is no significant difference between the measured and predicted values, which confirms the validation of the model.

### 3.2. Effect of Cooking on Total Polyphenol Content

**Figure 13:**
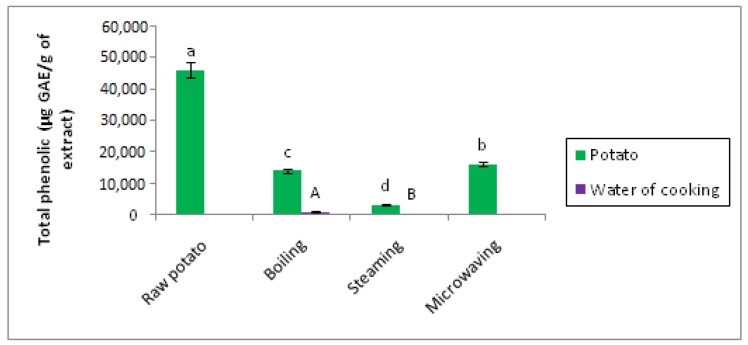
Effect of cooking on the total polyphenol content of potatoes. The superscript letters a, b, c, d, and e indicate a significant difference between raw and cooked potato at the 0.05 significance level. The superscript letters A, B indicate a significant difference between water of boiling and water of steaming at the 0.05 significance level.

There is a significant difference (*p* < 0.05) between the total polyphenol contents of raw potato, steam cooked, cooked in water, and microwaved (Figure 13).

These results indicate the richness of the potato in polyphenols, which has been proved by several studies [20,33,34,35]. The results obtained show that the three modes of cooking cause a decrease in the total polyphenol contents. The polyphenols are lost to different degrees according to the method of cooking, the classification of the polyphenol contents places the microwave in the first position, then comes the steam cooking and lastly the cooking in the water. The analysis of the cooking waters showed the presence of the phenolic compounds but at very low levels. The boiling water contains a high total polyphenol content relative to that obtained from steam cooking, and this is confirmed by the analysis of the variance where a significant difference was obtained between the two grades.

Several studies have shown that the cooking method reduces total phenolic content [20,21,36,37]. This decrease may be due to the thermal destruction of these compounds [17,38,39] and the solubilization of certain phenolic compounds in cooking water [40,41].

The chemical structure strongly influences the loss of total polyphenols, which may explain the differences between the three modes of cooking, as polyphenols are grouped into subclasses with common chemical skeletons, components of the same sub-class are affected differently by cooking. These variations can be explained by the hydroxylation scheme, sugar bonding, molecular size, polarity, and solubility of these compounds [21].

The decrease in polyphenol content during microwaving may be caused by the breakdown of weak hydrogen bonds by dipolar rotation of molecules [42]. This treatment accelerates the breakdown of the cells by causing a rapid increase in temperature and internal pressure in the walls of plant cells, which can lead to thermal destruction of polyphenols [43,44].

Referring to the bibliography, the results obtained on the effect of cooking on some phenolic compounds are contradictory. Several studies have confirmed that different cooking methods reduce phenolic compounds in potatoes, regardless of the cooking conditions: high cooking temperature levels, long duration, or a combination of factors [20], which is used to describe the temperature of the baking process [36,45].

Perla et al. [20] studied the impact of boiling and microwave cooking methods on potato phenolic compounds of five varieties with different skin and flesh colors after six months of storage. The level of phenolic compounds was reduced by both cooking methods, but boiling minimized these losses.

Mulinacci et al. [46] studied the effect of two cooking methods (boiling and microwave) on the phenolic and anthocyanin content of three varieties of potato.

Burgos et al. [47] showed that cooking of unpeeled potatoes did not cause any loss of phenolic compounds.

However, some authors have reported that cooking increases the content of these compounds, and this increase is attributed to the facilitated release of phenolic compounds previously bound to the cellular constituents during cooking. This release would compensate for any thermal degradation loss [48]. Navarre et al. [49] demonstrated that cooking in water lasting 18 min results in an increase in total polyphenol contents.

According to Faller and Fialho [22], cooking of the potato for 6.5 min increases the content of hydrolysable polyphenols. On the other hand it reduces the rate of soluble fractions. This result may explain the decrease in the phenolic acid and tannin content of the potato.

### 3.3. LC-MS/MS Analysis 

Changes in the contents of 27 components were measured after boiling, steaming and microwaving of potato (Table 6). LC-MS/MS analysis showed that the potato contains considerable amounts of tr-caffeic acid, apigenin, rhamnetin and chrysin. The cooking waters have small amounts of phenolic compounds. Analysis of the steaming water showed the presence of quinic acid, malic acid, chlorogenic acid, protocatechuic acid, tannic acid, and tr-caffeic acid with the appearance of two phenolic compounds which have not been detected in the plant matrix and are: 4-OH benzoic acid and quercetin. The identification and quantification of the phenolic compounds before and after cooking confirmed the spectrophotometric assay results. The three modes of cooking cause a decrease in the phenolic compounds; the microwave mode was the most effective in retaining the phenolic content. The cooking waters have small amounts of polyphenols. During the culinary treatment of potatoes, its intracellular structures undergo degradation, resulting in the release of phenolic acids from their non-soluble complexes [17].

The most poorly retained compounds were p-coumaric acid and rosmarinic acid, which were found in trace amounts in the case of cooking in water, and totally lost in the case of steam cooking. Although retention varied widely between polyphenols, many were affected in the same way by the three modes of cooking studied. The content of certain phenolic acids, chlorogenic acid, protocatechuic acid, tannic acid, and tr-caffeic acid remained almost stable during treatment despite their presence and solubilization in the cooking waters, this is probably due to their production by the breakdown of more complexpolyphenols [21].

A slight increase in coumarin after cooking and the appearance of two phenolic compounds in the steam cooking water that were not detected in the vegetable matrix (4-OH benzoic acid and quercetin) may be due to the release of the aglycones following the breakdown of more complex glycosides or esters during the treatment. In addition, certain phenolics may also be linked to non-digestible components of the food matrix, and breakage of the cellular structure by treatment can cause their release and solubilization [50].

These results are different from those obtained by Tudela et al. [17], which resulted in about 50% chlorogenic acid loss and Dao and Friedman [51], who showed that boiling decreased the amount of this acid–phenol ratio of 60%. The lowest changes in chlorogenic acid content were observed after microwave cooking, of −45% [36].

### 3.4. Antioxidant Activity

For the evaluation of the antioxidant activity, three methods were used. For each method two approaches are applied: on the one hand, determination of the relative reduction of DPPH, ABTS, and the reduction of copper, and determination, on the other hand, of the amount of antioxidant necessary to reduce 50% DPPH, ABTS, and Cu^2+^.

**Figure 14:**
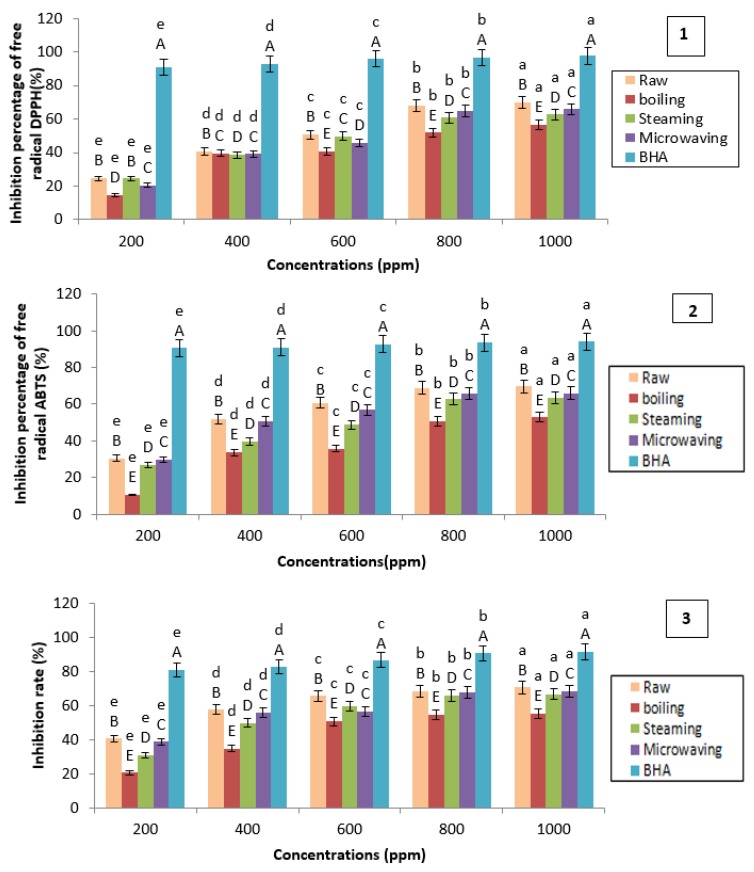
Evaluation of antioxidant activity: 1: 1,1-diphenyl-2-picryl-hydrazyl (DPPH) method; 2: 2,2′-azino-bis(3-ethylbenzothiazoline-6-sulphonic acid) (ABTS) method; 3: Cupric reducing antioxidant capacity (CUPRAC) method. BHA: Butylated hydroxyanisole. The superscript letters A, B, C, D, and E indicate a significant difference between antioxidant activities of raw and cooked potatoes at the same concentration of extract (ppm) (at the 0.05 significance level. The superscript letters a, b, c, d, and e indicate a significant difference between antioxidant activities of raw and cooked potatoes at different concentrations of extract (ppm) at the 0.05 significance level.

The antioxidant activities of raw and cooked (in water, steam, and microwave) potato extracts were tested and then compared to those of a reference antioxidant, Butylated hydroxyanisole (BHA). The results obtained for the three methods are presented in the Figure 14.

The results of the three methods of evaluating reductive activity expressed in I% clearly show that the potato extract has a moderate antioxidant power. The antioxidant activity increases with increasing concentration of the extract. This antioxidant activity was reduced by cooking to different degrees according to the cooking method. The classification of the cooking modes according to the degree of reduction of the antioxidant activity places the cooking in the microwave in the first position, then comes the steaming and, lastly, the cooking in water. The decrease in antioxidant activity may be related to the reduction of polyphenols by the various methods of cooking. Generally, the literature reports that there is a relationship between the content of phenolic compounds and antioxidant properties [52]. According to Hayes et al. [53] the antioxidant activity generally depends on the number and the position of the hydroxyl groups relative to the functional carboxyl groups. Montoro et al. [54] have shown that flavonoids are compounds exhibit high antioxidant activity. Caffeic acid (3,4-dihydroxycinnamic acid), one of the main hydroxycinnamic acids present in the potato extract, has been identified as one of the active antioxidants. Gulcin [55] evaluated the antioxidant activity of caffeic acid using different in vitro antioxidant tests, such as 2-azino-bis (3-ethylbenzthiazoline-6-sulfonic acid) (ABTS), antiradical activity of 1,1-diphenyl-2-picryl-hydrazyl (DPPH). The caffeic acid showed a potent antioxidant activity compared to the reference antioxidants (BHA, BHT, and α-Tocopherol).

These results are confirmed by the calculation of the parameter IC_50_ (Table 7). The results of ANOVA analysis showed a significant difference (*p* < 0.05) between the IC_50_s of raw and cooked potatoes (in water, steam, and microwave), and this has been observed for all the methods applied for the evaluation of the antioxidant activity.

From these results, it is noted that the IC_50_ of the cooked potatoes are higher than the IC_50_ of the raw potato. This indicates a decrease in antioxidant activity by the cooking process. The mode of cooking potatoes “in water” showed the highest IC_50_, i.e., the lowest antioxidant activity, followed by steaming and cooking in water. Potato IC_50_s are lower than IC_50_ BHA, meaning that raw and cooked potatoes have lower antioxidant activity than the reference antioxidant.

Similar results have also been obtained in previous studies.

According to Tian et al. [56], a moderate reduction in activity was observed after boiling and cooking in the microwave, while steaming reduced antioxidant activity by 21.57%.

Perla et al. [20] recorded significant decreases of 42.22% and 50.47% in the radical–antiradical activity due to boiling and microwaving.

Lemos et al. [57] also reported that cooking decreased the antioxidant activity of potatoes.

According to Faller and Fialho [22] cooking led to reductions in antioxidant capacity for most vegetables, with small differences between boiling, microwaving, and steaming. The polyphenols showed a positive correlation with antioxidant capacity in raw and cooked potatoes.

### 3.5. Anticholinesterase Activity

**Figure 15:**
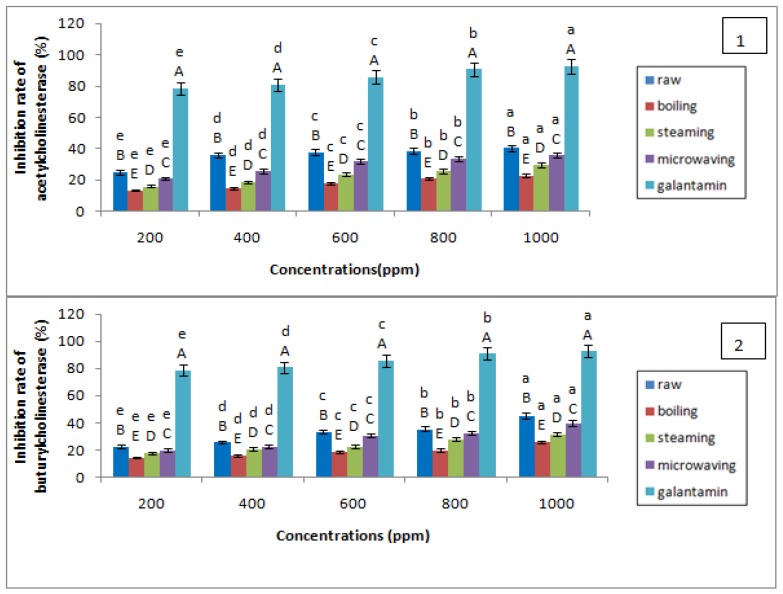
Evaluation of anticholinesterase activity of raw and cooked potato extracts: 1: antiacetylcholinesterase activity; and 2: antibutyrylcholinesterase activity. The superscript letters A, B, C, D, and E indicate a significant difference between anticholinesterase activities of raw and cooked potatoes at the same concentration of extract (ppm) (at the 0.05 significance level. The superscript letters a, b, c, d, and e indicate a significant difference between anticholinesterase activities of raw and cooked potatoes at different concentrations of extract (ppm) at the 0.05 significance level.

The antiacetylcholinesterase and antibutyrylcholinesterase activities of raw and cooked potato extracts (in water, steam, and microwave) were tested and then compared to that of a reference inhibitor, galantamine. The results obtained are presented in the Figure 15.

Potato extracts showed low inhibitory activity of acetylcholinesterase (AChE) and butyrylcholinesterase (BChE). A decrease in anticholinesterase activity was obtained after cooking, a significant difference (*p* < 0.05) exists between the raw potato and the cooked samples and the different concentrations of the extract.

This activity was decreased by cooking to different degrees according to the cooking method. The classification of the cooking modes according to the degree of reduction of the anticholinesterase activity places the microwave in the first position, then steam cooking and, lastly, cooking in water. From the values obtained, there is a significant difference (*p* < 0.05) between the raw potato, cooked potato, and the galantamine.

The raw and cooked potato extracts exhibit high IC_50_s for anti-acetylcholinesterase activity and antibuturylcholinesterase activity, indicating low anticholinesterase activity (Table 8). The IC_50_ obtained for the galantamine used asareference molecule is much lower than those of the extract, so galantamine has a better inhibitory activity of AChE and BChE. Galantamine is widely used as a reference substance in in vitro assays because of its strong inhibitory effect on AChE [58]. Today, it is authorized in several European countries as a treatment for AD in advanced stages [59].

The mode of cooking potatoes “in water” showed the highest IC_50_, i.e., the lowest anticholinesterase activity, followed by steaming and cooking in water.

Although most of the known potato inhibitors of the AChE enzyme are alkaloids [60] several studies have recently been carried out to identify other natural molecules that may have significant anti-AChE activity

Thus, according to Houghton et al. [61], several compounds, other than alkaloids, have a high ability to inhibit the AChE enzyme, such as terpenoids, phenolic compounds, flavonoids and isocoumarins. Several studies have shown strong anticholinesterase activity of phenolic compounds [62,63,64].

Several studies have shown the correlation between phenolic compounds and anticholinesterase activity. Several natural polyphenols have shown anticholinesterase effects [65]. In most in vivo studies, the anticholinergic activity of polyphenol was accompanied by an improvement in cognitive functions, such as learning and memory [66].

However, the exact mechanism of interaction with polyphenols in the cholinergic system is not yet clear [65].

Despite the richness of the potato raw and cooked in polyphenols, low anticholinesterase activity was obtained. This may be due to the nature and chemical structure, as well as the synergy and interactions between the polyphenols before and after cooking.

The number and position of the other hydroxyl groups in the molecules tested played a minor role in this context. Aglycones were the most effective cholinesterase inhibitors of their corresponding glycosylated forms. Overall, the results show that phenolic acids can play a role in neuroprotection [60].

Szwajgier [67] measured anticholinesterase activities of nine phenolic acids and six flavonoids, alone or in combination. The synergy/antagonism/lack of interaction between the compounds was evaluated taking into account the statistical significance. The modified spectrophotometric method of Ellman was used for the measurement of anticholinesterase activity. Anti-acetylcholinesterase activities were classified in this order: homogentisic acid > 4-hydroxyphenylpyruvic acid > nordihydroguaiaretic acid > rosmarinic acid > caffeic acid > acid gallic > homovanillic acid > sinapic acid. In most cases, a paired interaction of these phenolic compounds: *p*-hydroxyphenylpyruvic, caffeic, chlorogenic, gentisic, and homogentisic acids and nordihydroguaiaretic, rosmarinic acids have a lower inhibitory activity (*p* < 0.05) to the compound activity alone. In addition, interactions between phenolic acids in pairs with flavonoids (cyanidin, delphinidin, kaempferol, myricetin, phloridzine, pelargonidin, or quercetin), in most cases, have a lower inhibitory activity than that calculated separately for the two compounds (*p* > 0.05).

## 4. Conclusions

The results obtained in this study indicate that cooking has a negative impact on total phenolic content, antioxidant, and anticholinesterase activities of the Spunta variety of potato. Results obtained for the three cooking modes are different, which is due to the difference in the type of polyphenol bonds with the various components of potato matrix, extraction parameters, and analysis procedures. However, cooking in a microwave oven can be suggested as the best method of cooking to preserve phenolic compounds in these cases, as potato tissue is not placed in direct contact with hot water and the leaching of compounds in water is minimized.

## Figures and Tables

**Table 1:** Experiment factor levels for each cooking mode.

Factors	Raw Potato		Boiling		Steaming		Microwave	
	Level −1	Level +1	level −1	level +1	level −1	Level +1	level −1	Level +1
Cooking time	_	_	10 min	30 min	30 min	120 min	10 min	15 min
Power (watt)	_	_	_	_	_	_	400	800
Temperature of cooking	_	_	80 °C	100 °C	80 °C	100 °C	_	_
Volume of water of cooking	_	_	100 mL	500 mL	500 mL	1000 mL	10 mL	50 mL
Solvent concentration	60%	100%	60%	100%	60%	100%	60%	100%
Maceration time	1 h	24 h	1 h	24 h	1 h	24 h	1 h	24 h
Ultrasonication time	30 min	1 h	30 min	1 h	30 min	1 h	30 min	1 h
Grinding	No	Yes	No	Yes	No	Yes	No	Yes

Levels: minimum (−1) and maximum (+1).

**Table 2:** Confirmation results of the model with the measured values and the predicted values of the studied responses.

Points	Predicted Polyphenol Content (µg Gallic Acid Equivalent/g of Extract)	Measured Polyphenol Content (µg Gallic Acid Equivalent/g of Extract)
(60 min, 60 min)	4.667 × 10^4 a^	4.668 × 10^4 a^

The superscript letter a indicates a significant difference at the 0.05 significance level.

**Table 3:** Confirmation results of the model with the measured values and the predicted values of the studied responses.

Points	Predicted Polyphenol Content (µg GAE/g Extract)	Mesured Polyphenol Content (µg GAE/g of Extract)
(30 min, 91,9192 °C)	1416 × 10^4 a^	1.415 × 10^4 a^

The superscript letter a indicates a significant difference at the 0.05 significance level. GAE: Gallic Acid Equivalent.

**Table 4:** Confirmation results of the model with the measured values and the predicted values of the studied responses.

Points	Predicted Polyphenol Content (µg GAE/g of Extract)	Mesured Polyphenol Content (µg GAE/g of Extract)
(10 min, 80 °C)	3356.08 ^a^	3356.009 ^a^

The superscript letter a indicates a significant difference at the 0.05 significance level.

**Table 5:** Confirmation results of the model with the measured values and the predicted values of the studied responses.

Points	Predicted Polyphenol Content (µg GAE/g of Extract)	Measured Polyphenol Content (µg GAE/g of Extract)
(13.23 min, 448.48 Watts)	16,210 ^a^	16,208.99 ^a^

The superscript letter a indicates a significant difference at the 0.05 significance level.

**Table 6:** Liquid chromatography tandem-mass spectrometry (LC-MS/MS) analysis.

No.	Compound	Ion Parent (*m*/*z*)	MS2 (Collision Energy)	Quantification (µg Analyte/g of Extract)					
				RP	BP	SP	MP	WB	WS
1	Acid quinic	190.95	85 (22), 93 (22)	250.87	100.54	234.98	243.76	ND	42.67
2	Mallic acid	133.05	115 (14), 71 (17)	340.67	90.65	338.76	339.76	ND	34.76
3	Ferrulic Acid	172.85	85 (12),129 (9)	ND	ND	ND	ND	ND	ND
4	Gallic acid	169.05	125 (14), 79 (25)	ND	ND	ND	ND	ND	ND
5	Chlorogenicacid	353	191 (17)	14,500.87	4490.76	4497.87	4498.78	67.67	56.78
6	protocatéchic acid	152.95	109 (16), 108 (26	5500.66	5499.56	5500.21	5500.65	2500.78	49.78
7	Tannicacid	182.95	124 (22), 78 (34)	7032.373	7029.65	7030.76	7031.87	3500.78	49.98
8	Caféic acid	178.95	135 (15), 134 (24), 89 (31)	20,011.193	8907.87	9345.78	10,000.76	4500.78	52.78
9	Vanillin	151.05	136 (17), 92 (21)	ND	ND	ND	ND	ND	ND
10	*P*-coumarique Acid	162.95	119 (15), 93 (31)	7058.25	345.76	ND	540.76	ND	ND
11	rosmarinic Acid	358.9	161 (17), 133 (42)	8054.233	209.76	ND	7098.76	ND	ND
12	Hesperidin	611.1	303,465	ND	ND	ND	ND	ND	ND
13	Rutin	609.1	300 (37), 271 (51), 301 (38)	ND	ND	ND	ND	ND	ND
14	Hyperoside	463.1	300, 301	ND	ND	ND	ND	ND	ND
15	4-OH benzoïc acid	136.95	93, 65	ND	ND	ND	ND	ND	60.43
16	Salicylic acid	136.95	93, 65, 75	ND	ND	ND	ND	ND	ND
17	Myricetin	317	179, 151, 137	4657.87	367.76	4569.76	456.78	ND	ND
18	Coumarin	146.95	103, 91, 77	5098.76	567.78	5089.54	5088.76	ND	ND
19	Kaempferol	264.95	217, 133, 151	119.76	89.76	99.76	99.45	ND	ND
20	Quercetin	300.9	179, 151, 121	ND	ND	ND	ND	ND	56.87
21	Hesperetin	300.95	164, 136, 108	ND	ND	ND	ND	ND	ND
22	Naringinin	270.95	151, 119, 107	218 .123	217.99	218.06	218.16	ND	ND
23	Luteoléin	284.95	175, 151, 133	225.980	224.76	225	112.78	ND	ND
24	Fisetin	284.95	135, 121	ND	ND	ND	ND	ND	ND
25	Apigenin	268.95	151, 117	9452.76	450.76	9065.78	5687.78	ND	ND
26	Rhamnetin	314.95	165, 121, 300	8564.78	345.99	567.98	789.87	ND	ND
27	Chrysin	253	143, 119, 107	5456.65	234.78	709.78	4987.76	ND	ND

RP: raw potato; BP: boiling potato; SP: steaming potato; MP: microwaving potato; WB: water of boiling; WS: water of steaming; ND: no determined.

**Table 7:** Half-maximal inhibitory concentration IC_50_ values.

Extracts of Potato	DPPH	ABTS	CUPRAC
Raw	586.65 ± 0.76 ^d^	380.67 ± 0.23 ^d^	300.87 ± 0.26 ^d^
Boiling	703.54 ± 0.56 ^a^	686.76 ± 0.49 ^a^	592.78 ± 0.74 ^a^
Steaming	620.76 ± 0.89 ^b^	610.76 ± 0.67 ^b^	410 ± 0.25 ^b^
Microwaving	609.8 ± 0.23 ^c^	395.65 ± 0.98 ^c^	330.65 ± 0.14 ^c^
BHA	85.13 ± 0.45 ^e^	86.56 ± 0.34 ^e^	96.5 ± 0.09 ^e^

The superscript letters a, b, c, d, and e indicate a significant difference at the 0.05 significance level. DPPH: 1,1-diphenyl-2-picryl-hydrazyl; ABTS: 2,2′-azino-bis(3-ethylbenzothiazoline-6-sulphonic acid); CUPRAC: cupric reducing antioxidant capacity; BHA: Butylated hydroxyanisole.

**Table 8:** Half-maximal inhibitory concentration (IC_50_) values for anticholinesterase activity.

Extracts of Potato	Antiacetylcholinesterase Activity	Antibuturylcholinesterase Activity
Raw	1105.29 ± 0.24 ^d^	1087.84 ± 0.54 ^d^
Boiling	2471.27 ± 0.67 ^a^	2129.42 ± 0.21 ^a^
Steaming	1967.72 ± 0.98 ^b^	1611.22 ± 0.18 ^b^
Microwaving	1363.6 ± 0.56 ^c^	1234.68 ± 0.54 ^c^
Galantamin	84.23 ± 0.21 ^e^	86.56 ± 0.08 ^e^

The superscript letters a, b, c, d, and e indicate a significant difference at the 0.05 significance level.
